# A Study on Weak Edge Detection of COVID-19's CT Images Based on Histogram Equalization and Improved Canny Algorithm

**DOI:** 10.1155/2021/5208940

**Published:** 2021-10-28

**Authors:** Shou-Ming Hou, Chao-Lan Jia, Ming-Jie Hou, Steven L. Fernandes, Jin-Cheng Guo

**Affiliations:** ^1^School of Computer Science and Technology, Henan Polytechnic University, Jiaozuo 454000, China; ^2^CT Centre, Jiaozuo People's Hospital, Jiaozuo 454000, China; ^3^Department of Computer Science, Design & Journalism, Creighton University, Omaha, Nebraska, USA; ^4^Department of Thoracic Surgery, Jiaozuo Second People's Hospital, Jiaozuo 454000, China

## Abstract

The coronavirus disease 2019 (COVID-19) is a substantial threat to people's lives and health due to its high infectivity and rapid spread. Computed tomography (CT) scan is one of the important auxiliary methods for the clinical diagnosis of COVID-19. However, CT image lesion edge is normally affected by pixels with uneven grayscale and isolated noise, which makes weak edge detection of the COVID-19 lesion more complicated. In order to solve this problem, an edge detection method is proposed, which combines the histogram equalization and the improved Canny algorithm. Specifically, the histogram equalization is applied to enhance image contrast. In the improved Canny algorithm, the median filter, instead of the Gaussian filter, is used to remove the isolated noise points. The *K*-means algorithm is applied to separate the image background and edge. And the Canny algorithm is improved continuously by combining the mathematical morphology and the maximum between class variance method (OTSU). On selecting four types of lesion images from COVID-CT date set, MSE, MAE, SNR, and the running time are applied to evaluate the performance of the proposed method. The average values of these evaluation indicators are 1.7322, 7.9010, 57.1241, and 5.4887, respectively. Compared with other three methods, these values indicate that the proposed method achieves better result. The experimental results prove that the proposed algorithm can effectively detect the weak edge of the lesion, which is helpful for the diagnosis of COVID-19.

## 1. Introduction

COVID-19 has caused a health crisis worldwide, impacting all sectors of human life [1]. On 11 March 2020, COVID-19 was declared as an extremely high-risk disease by the World Health Organization (WHO) [2]. Up to April 16, 2021, more than 139.64 million people worldwide were infected, and more than 3 million people had died [3]. CT images contain a lot of important information, which can be used to evaluate disease quantitatively. In COVID-19 diagnosis, the reverse transcription-polymerase chain reaction (RT-PCR) detection is the most common way. However, when test kits are insufficient, especially during the outbreak phase, CT scan is a more effective diagnostic method. CT can show detailed symptoms of clinical diagnosis in COVID-19, especially for patients with moderate to severe [4]. Many hospitals use this technique to scan the lungs of patients and diagnose the illness. The severe acute respiratory syndrome coronavirus 2 (SARS-CoV-2) is determined as the cause of COVID-19 [5]. There are several CT image features of COVID-19, such as single ground-glass shadow, diffuse ground-glass shadow of both lungs, large area consolidation of both lungs, multiple patchy consolidations, and paving stone-like[6]. Edge detection algorithms are used to detect the lesion of CT images, which is helpful for doctors to diagnose the condition. However, the lesion will be missed or incorrectly detected due to poor contrast of the edge, thus affecting patient treatment. Therefore, weak edge detection is an urgent problem to be solved.

In recent years, weak edge detection has been researched by plenty of scholars. In 2012, Ji et al. [7] effectively extracted weak edge information in the skull using a combination of histogram equalization and mean-shift filter (called HMS from now on), but the edge continuity was flawed. In the same year, Xue et al. [8] developed the Laplace operator-based Hessian matrix to accurately detect blood vessels on uneven gray scale blood vessel images, which was only suitable for two-dimensional images. In 2015, Lin et al. [9] proposed a weak edge detection method based on the cumulative change rate, but this method required manual adjustment of the unified threshold. In 2016, Li et al. [10] segmented the prostate with the level set method from ultrasound images with weak edges. In 2017, Kathrin et al. [11] addressed the fuzzy edge detection of biomedical CT images by dynamic programming technology. In the same year, Khadidos et al. [12] proved that the weighted level set evolution method is an effective method in weak edge detection. In 2020, Wang and Xu [13] used fit methods to calculate new local terms of the variation segmentation model. The sensitivity of the method to the initial contour is reduced, and the accuracy of weak edge segmentation is improved. However, the segmentation process relies too much on manual experience. In the same year, Chetna et al. [14] designed an automatic segmentation technology based on energy curves, which used an automatic threshold to reduce step size, and then the breast cancer cells can be accurately identified.

From the experimental results, the above algorithms can extract weak edge, but there are still some defects, for example, the result is not so accurate of the COVID-19 CT image detection. In other words, the detection error needs to be further reduced. In this paper, based on histogram equalization and improved Canny algorithm, we propose an weak edge detection algorithm. Specifically, enhancing image edge is derived from capturing the details by using histogram equalization. Then, to obtain lesion edge, we embed median filter, *K*-means, mathematical morphology, OTSU algorithm into the Canny algorithm, and namely, the improved Canny algorithm. The proposed algorithm can efficiently eliminate noise of COVID-19 image and extract lesion's detail and edge.

The main contributions of this paper on weak edge detection of CT images are as follows:

1. The histogram equalization is combined with the improved Canny algorithm to establish an edge detection model. The model benefits from balancing the noise and the edge. In other words, the model can not only reduce the noise influence in COVID-19 CT images but also improve the accuracy of weak edge detection
2. The median filter is employed to optimize the Canny algorithm. Specifically, the median filter takes the place of the Gaussian filter so that the isolated noise point can be eliminated
3. In order to automatically obtain threshold, the novelty of this paper is using the OTSU algorithm

This paper is organized as follows.  Section 2 describes the data set applied by the proposed algorithm.  Section 3 depicts the related theories of the proposed algorithm, including histogram equalization, Canny algorithm, and the proposed algorithm. Experimental evaluation and comparison with other methods are discussed in Section 4. Concluding remarks are given in Section 5.

## 2. Dataset

**Figure 1:**
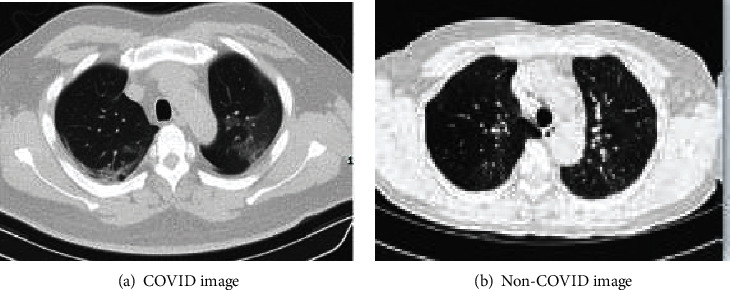
Example of CT images for COVID and non-COVID.

In this section, we introduce the dataset used in this paper. From the perspective of data authenticity, COVID-CT is used to verify the effectiveness of the algorithm [15], the dataset contains COVID and non-COVID, and Figure 1(a) and Figure 1(b) are representative image of COVID and non-COVID. From Table 1, COVID contains 349 CT images of clinical manifestation, which are from 216 patients, and the ratio of male to female is 86 : 51. And 463 CT images of healthy people are involved in non-COVID.

## 3. Methodology

In this section, we introduce the related methods, including histogram equalization, traditional Canny algorithm, the improved Canny algorithm, and the proposed method. Histogram equalization is detailed description firstly. Then, we improve the traditional Canny algorithm, namely, the improved Canny algorithm. Finally, we introduce the proposed method.

### 3.1. Histogram Equalization

Histogram equalization has been extensively used in image enhancement algorithms. The basic idea is that the original nonuniform probability distribution gray map of the CT image is nonlinearly stretched by the histogram and transformed into a uniform probability distribution map [16]. In other words, the image clarity is enhanced by adjusting the size of the gray value [17]. The theoretical formula of histogram equalization is as follows.

Calculate the normalized gray value of the original image and the normalized gray value of the mapped image, which are *r* and *s*, respectively. The relationship between *s* and *r* is as follows.

(1) $$\begin{array}{c} \text{s}=T\left(r\right)=\int_{0}^{r}P_{r}\left(r\right)dr, \end{array}$$

where *P*_*r*_(*r*) represents the probability density function of the image, and the range of *r*, *s*, and *T*(*r*) values is all in [0, 1]; also, *T*(*r*) increases monotonically in the interval.

Assume thatP_*s*_(*s*)is the probability density function after gray scale transformation, an the relationship betweenP_*r*_(*r*)andP_*s*_(*s*)is as follows.

(2) $$\begin{array}{c} {\left.P_{s}\left(s\right)=P_{r}\left(r\right)\frac{d_{r}}{d_{s}}\right|}_{r=T^{-1}\left(r\right)}. \end{array}$$

*B* is expressed as gray level, the image distribution probability is *P*(*r*_*B*_), the number of pixels is *n*_*B*_, the pixel is *N*, the total gray level is *L*, and the relationship is as follows.

(3) $$\begin{array}{c} {\text{P}}_{r}\left(B\right)=\frac{n_{B}}{N}, \end{array}$$

where *B* ∈ {0, *L*‐1}. When ∑_*B*=0_^*L*−1^*P*(*r*_*B*_) = 1, the image is a uniform probability density function.

**Figure 2:**
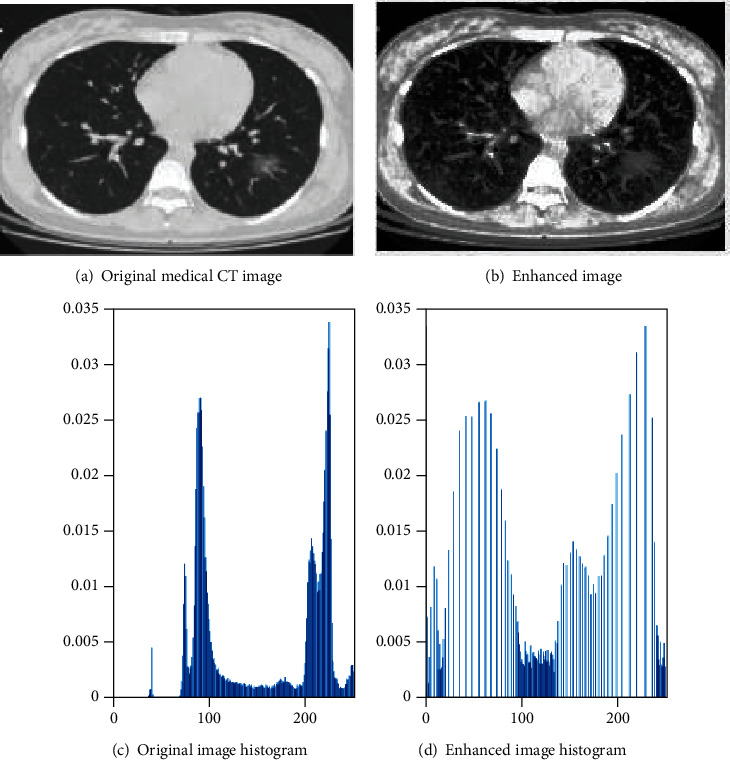
Comparison of the effect of histogram equalization algorithm.

Based on the histogram equalization algorithm, the single ground-glass shadow CT image features are enhanced, and the result is shown in Figure 2.  Figure 2(a) is the original medical CT image of the single ground-glass shadow, and Figure 2(c) is the original image histogram.  Figure 2(b) and Figure 2(d) are the enhanced original image and the enhanced histogram, respectively. We can observe that the weak pixels are enhanced, and the image details tend to be evenly distributed.

### 3.2. Traditional Canny Algorithm and Existing Problems

The edge detection is helpful towards analyzing CT images. At present, the mainstream edge detection algorithms include morphological processing [18], ant colony algorithm [19], watershed [20], Canny [21], and machine learning [22], in which Canny is one of the most widely used edge detection algorithms. This algorithm has the characteristics of high locating accuracy and effectively suppresses noise. The steps of the traditional Canny edge detection algorithm are as follows.

1. Gaussian filter. It can remove nonhigh frequency noise and smooth graph

(4) $$\begin{array}{c} G=\frac{1}{\sqrt{2\pi \sigma^{2}}}\exp\left(\frac{x^{2}}{2\sigma^{2}}\right), \end{array}$$

where *σ* means the standard deviation of Gaussian filter, and *G* represents the intensity of the pixel after smoothing.

(2) The gradient magnitude and direction. Traditional edge difference operator, such as Sobel, is used to calculate the gradient magnitude and direction, so as to get thick and bright image edge

(5) $$\begin{array}{c} G_{x}=\left[f\left(x+1,y-1\right)+2f\left(x+1,y\right)+f\left(x+1,y+1\right)\right]-\left[f\left(x-1,y-1\right)+2f\left(x-1,y\right)+f\left(x-1,y+1\right)\right],\\ G_{y}=\left[f\left(x-1,y-1\right)+2f\left(x,y-1\right)+f\left(x+1,y-1\right)\right]-\left[f\left(x-1,y+1\right)+2f\left(x,y+1\right)+f\left(x+1,y+1\right)\right], \end{array}$$

where *G*_*x*_ and *G*_*y*_ represent the gradient components in the horizontal and vertical direction, and *f*(*x*, *y*) represents the pixel value of the image.

Let *M* is the gradient magnitude, and the calculation formula of *G* is as follows.

(6) $$\begin{array}{c} M=\sqrt{G_{x}^{2}+G_{y}^{2}}. \end{array}$$

Assume that *J* represents the gradient direction, the calculation formula of *J* is as follows.

(7) $$\begin{array}{c} J=\arctan\left(\frac{G_{y}}{G_{x}}\right). \end{array}$$

(3) Nonmaximum suppression. After calculating the gradient magnitude and direction, the sharpest position is retained to ensure a clearer boundary in the gradient intensity change. Comparison of gradient intensity between current and other directions, the image boundary point is known as the current is greater than the other directions of gradient intensity. Otherwise, the gradient intensity threshold is set to 0, which can eliminate this point. Suppose *P* is the gradient intensity of the current pixel, *P*_1_ and *P*_2_ are the gradient intensity in positive and negative directions, respectively. The pseudocode for nonmaximum suppression is as follows

(4) Dual-threshold and hysteresis boundary track. Dual-threshold means setting high and low thresholds. The high threshold is greater than the pixel gradient value, and it is a strong edge and namely image edge. The low threshold is less than the pixel gradient value, and it is considered not to be the edge of the image and discarded. Weak edge points are defined as the pixel value varies between high and low thresholds. Finally, hysteresis boundary tracking is applied to determine whether the weak edge is an image edge

The traditional Canny algorithm may causes some problems while detecting the edge of the image.

First of all, the variance of Gaussian filtering determines the image smoothing effect and noise removal effect. Generally speaking, the size of the variance is fixed. If the variance is too large, Gaussian filtering will help to remove noise, but it will cause serious loss of image detail, resulting in blurred image edge; if the variance is too small, the denoising effect of Gaussian filtering will be poor.

Secondly, the Sobel operator is used to calculate the gradient amplitude and direction, and the obtained edge is relatively thick.

Finally, the dual-threshold detection method will generate more false edge, which will break the target edge and lose the edge information.

### 3.3. Improved Canny Algorithm

We use the median filter, *K*-means algorithm, mathematical morphology, and OTSU algorithm to improve the classical Canny algorithm.

**Figure 3:**
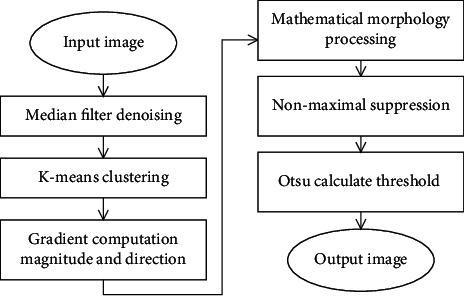
Improved Canny algorithm.

Figure 3 is a flowchart of the improved Canny algorithm. Firstly, the Gaussian filter in the Canny algorithm is modified to the median filter. Next, based on the *K*-means, the Sobel operator is utilized to calculate the gradient magnitude and direction. Then, mathematical morphology is applied to thin out the edge. Finally, after nonmaximum suppression processing, the OTSU algorithm is used to determine the threshold automatically. The steps of improvement are as follows.

**Figure 4:**
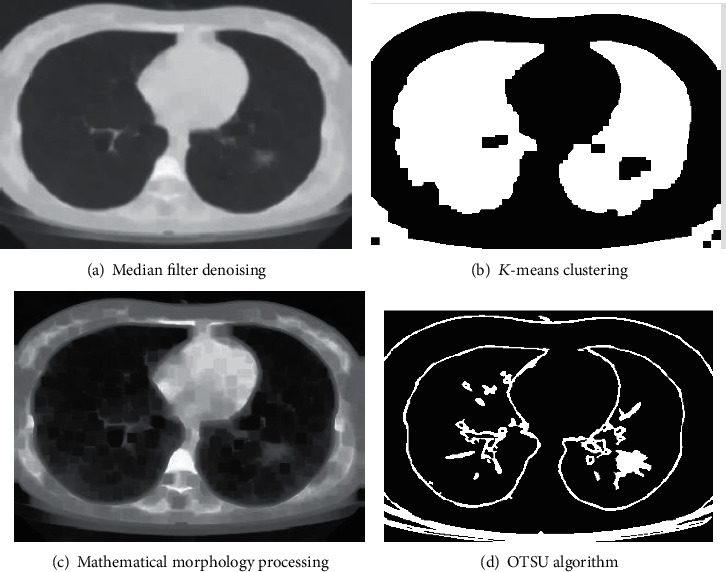
Experimental result images.

The experimental results obtained after median filter, *K*-means clustering, mathematical morphology, and OTSU algorithm are shown in Figures 4(a)–4(d). We can observe that after processing by several methods, the edge of the acquired lesion is very clear.

#### 3.3.1. Median Filter

COVID-19 CT image is complicated to understand; so, we need to remove undesirable portions, including noise [23]. The isolated noise points are eliminated by the median filter [24]. The median filter arranges the pixel values in the neighborhood of a point from large to small and takes the median value as the new pixel value to output, thereby eliminating isolated noise points. The algorithm is as follows.

1. Sort all elements in the array *ρ* = [*ρ*_11_, *ρ*_12_, *ρ*_13_, *ρ*_21_, *ρ*_22_, *ρ*_23_, *ρ*_31_, *ρ*_32_, *ρ*_33_] from large to small

(8) $$\begin{array}{c} \rho =\text{med}\left\{\rho_{1\varphi },\rho_{2\varphi },\rho_{3\varphi }\right\}, \end{array}$$

where *φ* = [1, 2, 3]. The commonly used sorting templates are 3 × 3 and 5 × 5. In this paper, 3 × 3 template is cited for processing. The original pixel template is shown in Table 2.

(2) Take the value of each row for comparison and obtain the value of each row maximum, intermediate, and minimum, which are Max_*µφ*_, Med_*µφ*_, and Min_*µφ*_, respectively. At this time, the pixel template is shown in Table 3

(3) Compare the data in each column and get the maximum value, respectively, which is Max_1_, Max_2_, and Max_3_. Finally, by comparing the Max_1_, Max_2_, and Max_3_, the middle value is result

#### 3.3.2. *K*-Means Algorithm

*K*-means algorithm is one of the most commonly applied clustering algorithms. By randomly selecting *K* points as the starting center point, then finding the same characteristic data and letting it be grouped into the same cluster [25]. Cluster ensures that between similar sample points are compact, and between different types of samples is scattered so that the clustering effect is optimal.

The main flow of the algorithm is as follows.

1. Given the sample data *w*, the set of objects is *A* = {*w*_1_, *w*_2_ ⋯ ⋯, *w*_*n*_}, and the initialization center *B* = {*C*_1_, *C*_2_ ⋯ ⋯, *C*_*k*_}

According to the principle of the minimum norm of grey value, the sample data point (*w*, *v*) is assigned to the cluster closest to it. The calculation formula is as follows.

(9) $$\begin{array}{c} U_{i}=\min\left|f\left(w,v\right)-C_{i}\right|, \end{array}$$

where *U*_*i*_ is the minimum value of the distance between the sample point *f*(*w*, *v*) and the various centroids *C*_*i*_.

(2) Calculate the cluster center point again and use it as the new cluster center point *T*_*i*_, *i* = 1, 2 ⋯ ⋯*k*. Formula is as follows

(10) $$\begin{array}{c} T_{i}=\frac{\sum_{i‐1}^{m}\left\{C_{i}=j\right\}x_{i}}{\sum_{i‐1}^{m}\left\{C_{j}=j\right\}}. \end{array}$$

(3) Repeat steps 2 and 3 until it reaches the maximum number of iterations

#### 3.3.3. Mathematical Morphology

The basic idea of mathematical morphology is using structural elements to obtain graphical shapes [26]. The main processing processes include expansion, erosion, reconstruction, opening operation, filling, closing operation, and edge extraction. The most basic operations are expansion and corrosion. The most typical operations combining the two are opening and closing operations.

Let *T* represents an image, *τ*(*α*, *β*) is the gray value at the point (*α*, *β*), g(*σ*, *ε*) is the structural element of the image, the domains of *τ*(*α*, *β*) and g(*σ*, *ε*)are *D*_*τ*_ and *D*_*b*_, and (*α*‐*σ*, *β*‐*ε*) ∈ *D*_*τ*_, (*α* + *σ*, *β* + *ε*) ∈ *D*_*b*_, and (*σ*, *ε*) ∈ *D*_*b*_.

Expansion is used to fill the voids in the graphics and expand the boundaries of the graphics outwards. The calculation formula is as follows.

(11) $$\begin{array}{c} \left(\tau ⊕\text{g}\right)\left(\alpha ,\beta \right)=\max\left\{\tau \left(\alpha ‐\delta ,\beta ‐\varepsilon \right)+g\left(\delta ,\varepsilon \right)\right\}, \end{array}$$

where ⊕ indicates the expansion operation. The expansion operation is the maximum value of *τ* ⊕ g in the domain determined by the structural elements.

Corrosion is used to eliminate small, useless edges, and shrink the image boundary inward. The calculation formula is as follows.

(12) $$\begin{array}{c} \left(\tau \Theta \text{g}\right)\left(\alpha ,\text{y}\right)=\min\left\{\tau \left(\alpha +\delta ,\beta +\varepsilon \right)-g\left(\delta ,\varepsilon \right)\right\}, \end{array}$$

where Θ represents the corrosion operation, which is the minimum value of *τ*Θg in the domain determined by the structural elements.

The step of the open operation is to corrode first and then expand. It can smooth the contour and filter out isolated parts that noncontain structural elements. The calculation formula is as follows.

(13) $$\begin{array}{c} \tau \circ \text{g}=\left(\tau ⊕\text{g}\right)\Theta \text{g}. \end{array}$$

The step of the closed operation is to expand first and then corrode. It can fill in small holes and connect the image boundaries to make them smooth. The calculation formula is as follows.

(14) $$\begin{array}{c} \tau \cdot \text{g}=\left(\tau \Theta \text{g}\right)⊕\text{g}, \end{array}$$

where ∘ represents the open operation, and · represents the closed operation.

Due to the shrinkage of the open operation and the expanding ability of the closed operation, the results will be biased. In this paper, using equation (15), it is an average method to eliminate the influence of offset.

(15) $$\begin{array}{c} T=\frac{1}{2}\left(\tau \circ \text{g}+\tau \cdot \text{g}\right). \end{array}$$

#### 3.3.4. OTSU Algorithm

The maximum between-class variance algorithm, also known as the OTSU algorithm, is an algorithm used to automatically determine the image binarization threshold. Because of its simple calculation and not affected by image contrast and brightness, it is widely used in global threshold determination [27]. The basic idea is: according to the gray-scale characteristics of the image, and the image is divided into two parts: the foreground and the background. The difference of value is greater between the foreground and the background, and the effect is better. Based on the input image, the OTSU algorithm automatically calculates the threshold value [28]. In this paper, we firstly set the threshold as 1, then, using the maximum interclass variance to calculate the optimal threshold.

Let *γ* represent the pixel gray value of a certain point in the image, [0, *R*] is the pixel gray level, and *n*_*γ*_is the number of pixels; thus, the value of the total number of pixels *N* is as follows.

(16) $$\begin{array}{c} N=\overset{R}{\underset{\gamma =0}{\sum }}n_{\gamma }. \end{array}$$

The probability of each pixel at gray level *γ*:

(17) $$\begin{array}{c} P_{\gamma }=\frac{n_{\gamma }}{N}. \end{array}$$

The threshold is used to divide the image pixels into two parts: *Q*_1_ and *Q*_2_; then, the probabilities of *Q*_1_ and *Q*_2_ at [0, *L* − 1]_,_ and [L‐1, *R*] are *D*_1_ and *D*_2_, and *D*_1_ + *D*_2_ = 1.

(18) $$\begin{array}{c} D_{1}=\overset{L-1}{\underset{\gamma =0}{\sum }}P_{\gamma },\;D_{2}=\overset{R}{\underset{L}{\sum }}P_{\gamma }. \end{array}$$

The mean values of *Q*_1_ and *Q*_2_ are *m*_1_ and *m*_2_, respectively, and *m*_*θ*_ is the global mean value of the image.

(19) $$\begin{array}{c} m_{\theta }=D_{1}\times m_{1}+D_{2}\times m_{2} \end{array}$$

The between-class variance expression is as shown in formula (20).

(20) $$\begin{array}{c} \sigma^{2}=D_{1}\times {\left(m_{1}‐m_{\theta }\right)}^{2}+D_{2}\times {\left(m_{2}‐m_{\theta }\right)}^{2}. \end{array}$$

### 3.4. The Proposed Method

**Figure 5:**
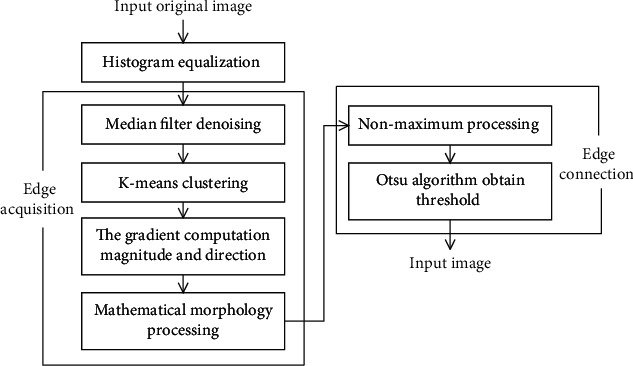
Algorithm flow chart.

In this paper, the proposed weak edge detection method of CT image is mainly divided into three steps. Firstly, enhancing the image detail feature by histogram equalization, secondly, obtaining the weak edge of the lesion on CT image by using median filter, *K*-means, Sobel operator, and mathematical morphology, and thirdly, combining nonmaximum suppression and OTSU algorithm to connect the edge. The flow of the proposed algorithm is shown in Figure 5.

Specific steps are as follows:

1. Histogram equalization. Performing histogram equalization on the grayscale image to enhance image detail
2. Edge acquisition. Firstly, using the median filter to preserve the grayscale characteristic of the image and reduce the noise of the image. Then, the *K*-means algorithm is used to classify the edge of the image for obtaining edge points and nonedge points. After calculating the gradient magnitude and direction, a thick and bright edge is obtained, which further enhances the brightness of the weak edge. Finally, the edge is refined using mathematical morphology to obtain more accurate edge
3. Edge connection. After nonmaximum value processing, the OTSU algorithm is applied to automatically obtain the best threshold and connect weak edge

### 3.5. Measure

In order to objectively evaluate the performance of the Canny algorithm, the HMS algorithm, the ETAR algorithm (Benhamza and Seridi [29] proposed algorithm, called ETAR from now on), and the proposed algorithm, we select mean square error (MSE), signal-noise ratio (SNR), mean absolute error (MAE), and the running time. MSE is used to evaluate the deviation between the observed value and the true value. The value of MSE is larger, and the detection error is greater. SNR refers to the false detection probability of nonedge points. If the value of SNRis larger, indicating that the false detection rate is lower, the detection effect is better. MAE represents the average of the error between the predicted value and the true value. The value of MAE is smaller, which means the error is smaller and the fusion effect is better. The calculation formulas of MSE, SNR, and MAE are as follows.

(21) $$\begin{array}{c} \text{MSE}\left(I_{H},I_{W}\right)=\frac{1}{MN}{\left(I_{H}\left(i,j\right)-I_{W}\left(i,j\right)\right)}^{2},\\ \text{SNR}=\log\frac{\sum_{i=1}^{M}\sum_{j-1}^{N}{\left(I_{w}\left(i,j\right)\right)}^{2}}{\sum_{i=1}^{M}\sum_{j=1}^{N}{\left(I_{H}\left(i,j\right)-I_{w}\left(i,j\right)\right)}^{2}},\\ \text{MAE}\left(I_{H},I_{W}\right)=\frac{1}{MN}\left(I_{H}\left(i,j\right)-I_{W}\left(i,j\right)\right), \end{array}$$

where *I*_*H*_ and *I*_*W*_ represent the fused image and the ideal image, respectively, and *I*_*H*_ and *I*_*W*_ are the pixel gray values of the fused image and the ideal image in the *i*th row and *j*th column, respectively.

## 4. Results and Discussion

**Figure 6:**
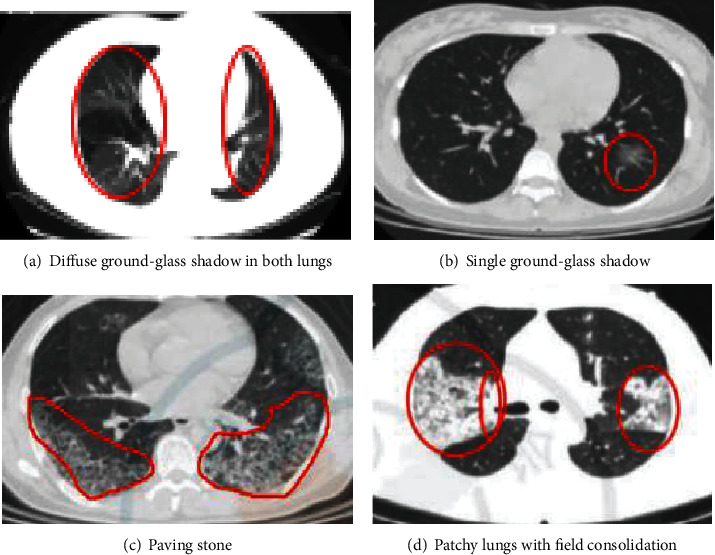
Partial sample data.

In this section, firstly, we introduce selected four types of CT lesion images. Then, by MSE, SNR, MAE, and the running time, we verify performance of the proposed algorithm in these images. And the results are shown in the table and figure below. Specifically, we intend to compare the performance of the proposed algorithm with the following algorithms, including the Canny algorithm, the HMS algorithm, and the ETAR algorithm. In the experiment, we use the COVID-CT for verifying the effect of the proposed algorithm on the denoising ability and weak edge detection. Four types of typical sample images selected from the dataset are shown in Figure 6.  Figure 6(a) is diffuse ground-glass shadow in both lungs, and the lesion tissue is relatively unclear.  Figure 6(b) is single ground-glass shadow, Figure 6(c) is paving stone, and their lesion's edge is relatively weak.  Figure 6(d) is patchy lungs with field consolidation, and the lesion's tissue is clear, but it is tightly connected with the lung tissue.

### 4.1. Verification of the Detection Effect of Diffuse Ground-Glass Shadow in Both Lungs

**Figure 7:**
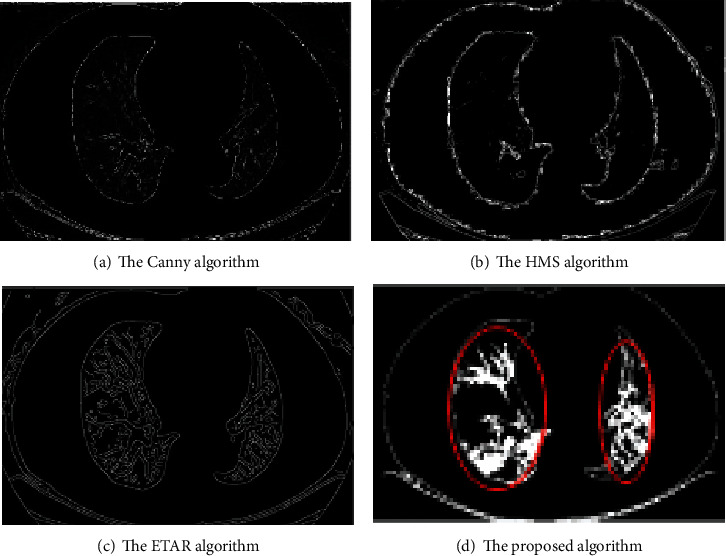
Comparison of the detection of diffuse ground-glass shadow in both lungs.

Compared the edge detection effect of diffuse ground-glass shadow in both lungs, the results are shown in Figure 7. The boundary of the lesion detected by the Canny algorithm is not continuous. The HMS algorithm detection result image contains a lot of noise, and the lesion's detail cannot be detected. The ETAR algorithm can detect the lesion, but the detected lesion feature is different from the actual lesion feature. The proposed algorithm not only effectively removes noise but also clearly shows the characteristics of the lesion.

The MSE, SNR, and MAE of diffuse ground-glass shadows in both lungs are shown in Table 4. The MSE is 1.0513 of the proposed algorithm, and the MSE of other algorithms exceeds 1.1000, which means that the detection error of the proposed algorithm is the smallest. From the SNR values of the four algorithms in Table 4, it can be seen that the SNR of the proposed algorithm is the smallest, indicating detection error is the smallest. The MAE is 65.0062, but the MAE values of all other algorithms exceed 150.0000, suggesting that the average error of the proposed algorithm between the predicted value and the true value is the smallest, and the detection effect is the best.

### 4.2. Verification of the Detection Effect on Single Ground-Glass Shadow

**Figure 8:**
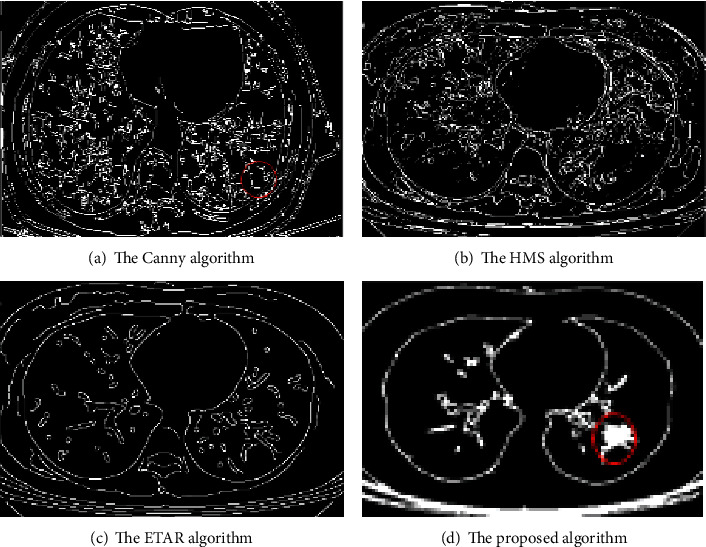
Comparison of single ground-glass shadow detection.

Figure 8 shows the experimental results of four algorithms for detecting the edge of single ground-glass shadow. We found that the denoising effect and the accuracy of the detected lesion features of the proposed algorithm are better than other three algorithms.

In the experiment, we use three data evaluation indicators to verify the detection effect of the proposed algorithm. We can see from Table 5 that the detection effect of the proposed algorithm is significantly better than the other algorithms. From a data point of view, the MSE, SNR, and MAE of the proposed algorithm are 2.8982, 7.0879, and 55.1343, respectively, while the MSE and MAE of the other algorithms are greater than 5.000 and 150.0000, and the SNR is less than 7.0000, respectively.

### 4.3. Verification of Detection Effect on Paving Road Shape

**Figure 9:**
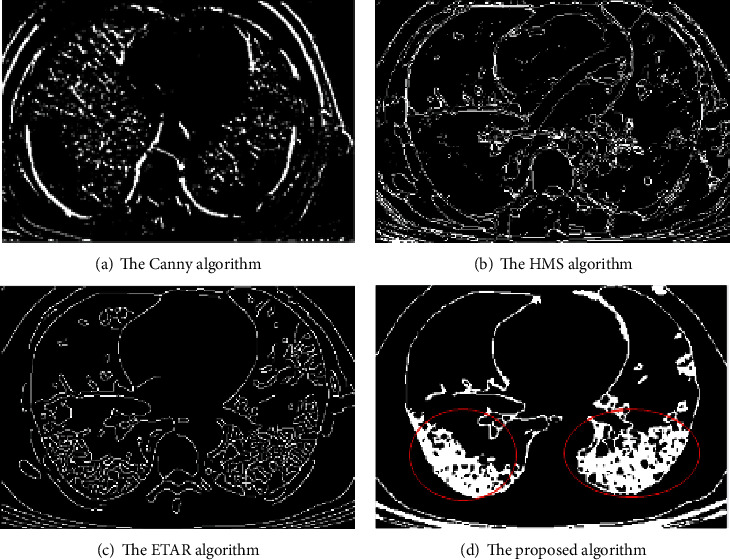
Comparison diagram of paving road shape detection.

For paving road CT image, we compare the edge detection effect of the proposed algorithm with the other three algorithms. The detection effect images are shown in Figure 9. We can see that the lesion feature detected by the proposed algorithm is more clear.

The experimental results are shown in Table 6. After analysis, we can conclude that the experimental results of the proposed algorithm are significantly better than the other three algorithms, which proves that the detection error of the algorithm is the smallest and the detection effect is the best.

### 4.4. Verification of Detection Effect on Patchy Lungs with Field Consolidation

**Figure 10:**
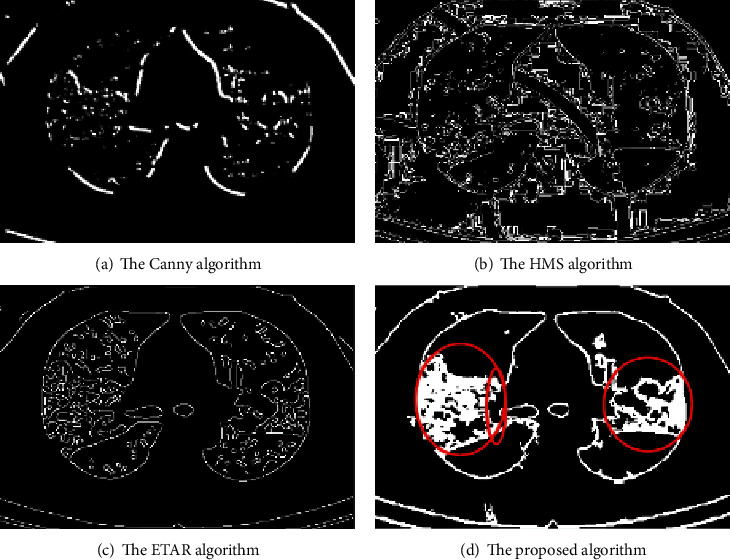
Comparison of the detection of patchy lungs with field consolidation.

To verify the effect of edge detection, the Canny algorithm, the HMS algorithm, the ETAR algorithm, and the proposed algorithm are used to carry out experiments on patchy lungs with field consolidation. The result is shown in Figure 10, compared with the other three algorithms, the proposed algorithm has better noise robustness, and the detected lesion feature is more accurate.

In order to evaluate the performance of the proposed algorithm, a comparative analysis is carried out from quantitative perspective.  Table 7 shows the data of the four algorithms under the three evaluation indicators. The experimental results show that the proposed algorithm using histogram equalization and improved Canny algorithm has the best denoising effect and the highest accuracy of lesion detection.

### 4.5. The Running Time

**Figure 11:**
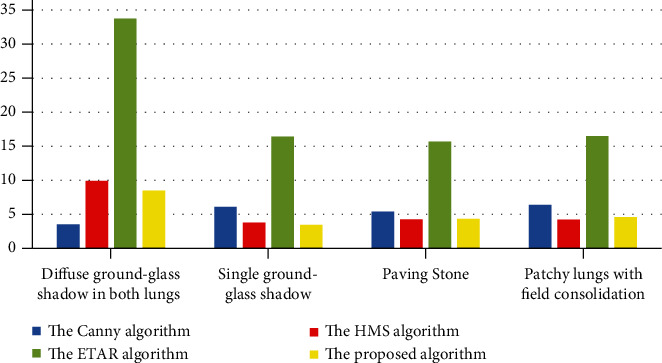
The running time of cases.

In order to further intuitively evaluate the edge detection effect of the Canny algorithm, the HMS algorithm, the ETAR algorithm, and the proposed algorithm, the edge detection time is calculated of the above cases.  Figure 11 gives the result; in terms of the running time of all images, we can observe that the proposed algorithm is shorter as compared to other three algorithms. Therefore, the proposed algorithm is considered to be one of the best edge detection algorithms.

## 5. Conclusion

In this paper, aiming at the weak edge problem in the edge detection of lesion from COVID-19 CT image, a fusion algorithm is proposed which includes three improvements: (1) usage of histogram equalization, we can obtain the maximum entropy of the image, thereby improving the clarity of weak edge. (2) The median filter, *K*-means algorithm, and mathematical morphology are added to the Canny algorithm, which can make the edge of the inspected more accurate. (3) The OTSU algorithm is used to automatically obtain the best threshold. Compared with the Canny algorithm, the HMS algorithm, and the ETAR algorithm, these improvements enable our method to have improved performance. The average values of MSE, MAE, SNR, and the running time of four types of lesion images are 1.7322, 7.9010, 57.1241, and 5.4887, respectively, indicating that our method has better results. Experiments show that the proposed algorithm can effectively extract the weak edge of the lesion.

In the future, we can do further research from the following two aspects: (1) The average running time is 5.1887 seconds, and we needs to further reduce the average running time for the purpose of improving the performance of the proposed method. (2) In order to improve the credibility of the experiment, we will carry on experiment on more data set.

## Figures and Tables

**Pseudocode 1:**
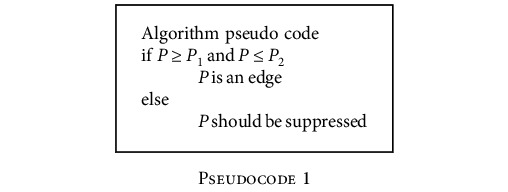


**Table 1:** Comparison of COVID and non-COVID.

	Images	Patients	Male/female
COVID	349	216	86/51
Non-COVID	463	55	

**Table 2:** the original pixel template.

	Column 1	Column 2	Column 3
Row 1	*g*_11_	*g*_12_	*g*_13_
Row 2	*g*_21_	*g*_22_	*g*_23_
Row 3	*g*_31_	*g*_32_	*g*_33_

**Table 3:** Pixel template after processing.

	Column 1	Column 2	Column 3
Row 1	Max_11_	Med_12_	Min_13_
Row 2	Max_21_	Med_22_	Min_23_
Row 3	Max_31_	Med_32_	Min_33_

**Table 4:** Comparison of detection indexes of diffuse ground-glass shadow in both lungs.

	The Canny algorithm	The HMS algorithm	The ETAR algorithm	The proposed algorithm
MSE	1.1230	8.2723	1.3313	1.0513
SNR	0.0457	0.0475	1.4373	6.6078
MAE	155.2991	157.5637	196.9864	65.0062

**Table 5:** Comparison of single ground-glass shadow lesion detection indicators.

	The Canny algorithm	The HMS algorithm	The ETAR algorithm	The proposed algorithm
MSE	5.8147	5.6171	8.3632	2.8982
SNR	2.0091	4.3257	2.3107	7.0879
MAE	157.2183	176.4992	158.5642	55.1343

**Table 6:** Comparison table of paving road detection indicators.

	The Canny algorithm	The HMS algorithm	The ETAR algorithm	The proposed algorithm
MSE	9.2358	9.3340	9.3341	1.8103
SNR	0.0460	3.8837	2.7741	7.4247
MAE	173.6431	172.6516	174.6519	67.1646

**Table 7:** Comparison table of detection indexes for patchy lungs with field consolidation.

	The Canny algorithm	The HMS algorithm	The ETAR algorithm	The proposed algorithm
MSE	1.3192	5.617	1.3313	1.1690
SNR	0.0397	3.2443	2.8746	10.4839
MAE	195.9766	174.4995	196.9860	41.1912

## Data Availability

The data used to support the findings of this study are available from the corresponding author upon request.
